# In vitro development of the Autonomous Colonoscope Robot System (ACRS) for fully automated colonoscope insertion

**DOI:** 10.1038/s43856-026-01747-8

**Published:** 2026-06-30

**Authors:** Keiichiro Kume, Tatsuru Taira, Seigo Terao, Nobuo Sakai

**Affiliations:** 1 Third Department of Internal Medicine, University of Occupational and Environmental Health, Japan, School of Medicine, Kitakyushu, Japan; 2 Department of Mechanical and Control Engineering, Faculty of Engineering, Kyushu Institute of Technology, Kitakyushu, Japan

**Keywords:** Colonoscopy, Cancer screening

## Abstract

**Background:**

In the field of colonoscopy, robotic systems have been developed to support or replace human operators due to a shortage of trained endoscopists. We developed the Autonomous Colonoscope Robot System (ACRS), based on the Endoscopic Operation Robot version 4, to evaluate whether expert-derived operational data can enable autonomous colonoscope insertion.

**Methods:**

ACRS was trained using insertion data obtained from an expert endoscopist operating a standardized colonoscopy training model. Automated insertions were evaluated using Pattern 1 of the model, a highly controlled configuration without substantial loop formation. Completely automated insertions were designated Level 4, whereas insertions requiring some manual assistance were designated Level 3. Level 4 insertion times were compared with manual insertions performed by an expert and trainees.

**Results:**

Of the 72 automated insertions at Level 3 or higher, 62 are classified as Level 4, giving a success rate of 86.1% (95% CI, 75.9–93.1%). The average insertion time for Level 4 procedures is 2.92 ± 1.20 minutes, significantly longer than that of the expert (1.43 ± 0.32 minutes), but comparable to the time taken by trainees (2.97 ± 1.32 minutes; errors are standard deviations).

**Conclusions:**

ACRS demonstrates proof-of-concept feasibility for autonomous colonoscope insertion under simplified, controlled model conditions. Further validation in more complex models, animal studies, and clinical settings is required before translational application.

## Plain language summary

Colonoscopy is an important test for finding colorectal cancer early. An endoscope is a thin, flexible tube with a camera that is inserted into the colon, but safe and painless insertion requires considerable skill. We developed the Autonomous Colonoscope Robot System (ACRS), based on a master–slave robotic system, in which a doctor’s hand movements are transmitted to a robotic device that holds and moves the endoscope. Using insertion data from an expert doctor in a colonoscopy training model, we trained an artificial intelligence model to control the robot automatically. In a simplified model setting, ACRS achieved fully automated insertion in many trials. This proof-of-concept study may support future development of robotic systems that help make colonoscopy safer and more widely available.

## Abstract (web summary)

Kume et al. develop an autonomous robotic system for colonoscope insertion using expert-derived operational data. The system achieves proof-of-concept automated insertion in a simplified colonoscopy training model, supporting further stepwise validation.

## Introduction

Colorectal cancer is the third most common cancer worldwide and the second leading cause of cancer-related death^1^. The gold standard for early detection of colorectal cancer is colonoscopy, which is performed approximately 19 million times annually in Europe and the United States^2^, and it has been shown to reduce both the incidence and mortality of colorectal cancer^3^. However, pain-free insertion of a colonoscope requires advanced training, and a large number of skilled endoscopists need to be trained. Currently, there are not enough practitioners to meet the demand^4,5^. To overcome this problem, several groups have undertaken the development of robotic colonoscopy systems^6,7^, with two autonomous insertion robots in particular attracting attention. One of these systems is the Aer-O-Scope (GI View Ltd., Ramat-Gan, Israel), which uses a balloon-based insertion mechanism resembling that of the double-balloon enteroscope. It achieves fully automated insertion through an inchworm-like motion generated by the inflation and deflation of balloons mounted at the distal ends of both the scope and its outer sheath. The system reached commercial availability, but was withdrawn from the market in its early stages^8^. Another system, called the Magnetic Flexible Endoscope, features a catheter-like tube with a camera at its tip, which is navigated autonomously via an external magnetic arm. To date, this system has only been tested in live pigs^9^. However, due to the nature of the propulsion mechanisms specific to these systems, both of which are primarily designed for insertion operations, they struggle with navigating complex and variable colonic anatomies, including cases where insertion is difficult. Moreover, these systems are not well-suited for handling the detailed observation required to detect small lesions hidden in blind spots behind colonic folds. These precise operations currently remain achievable only through manual colonoscopy performed directly by an endoscopist^10,11^.

In light of this situation, the Endoscopic Operation Robot (EOR), a master-slave robotic system for robotic insertion of a colonoscope, was developed. The EOR has a colonoscope (PCF-240, Olympus, Tokyo, Japan) mounted on the slave unit, and insertion and retraction, rotation, and angulation can be intuitively controlled using a proprietary master unit^12–14^. In the present study, we develop the Autonomous Colonoscope Robot System (ACRS) based on EOR ver.4 and evaluate its ability to perform automated colonoscope insertion using Pattern 1 of a colonoscopy training model (Kyoto Kagaku Co., Ltd., Kyoto, Japan). ACRS achieves fully automated insertion in a high proportion of trials under simplified and controlled model conditions, and its insertion time is comparable to that of trainees. These findings demonstrate the proof-of-concept feasibility of autonomous colonoscope insertion using expert-derived operational data, while also indicating that further validation in more complex models, animal studies, and clinical settings is required before translational application can be considered.

## Methods

After an overview of the specifications of EOR ver.4, the AI model developed for autonomous insertion and the mechanism by which autonomous operation is achieved are described. The method used to validate the fully automated colonoscope insertion by ACRS is then presented. This validation was conducted using Insertion Pattern 1 of a colonoscope training model.

### Development history and specifications of EOR ver.4

**Fig. 1:**
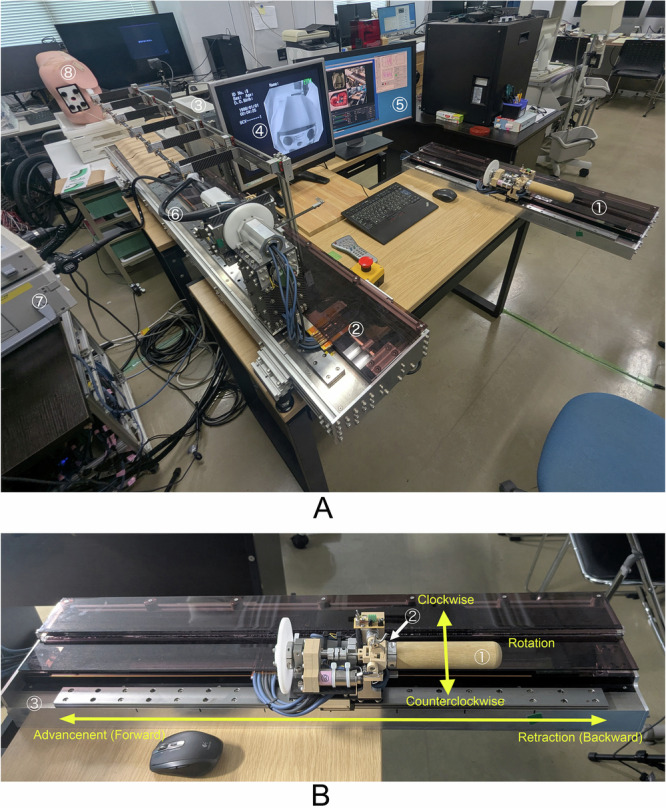
Endoscopic Operation Robot (EOR) version 4 and master unit. **A** EOR version 4 and the colonoscopy training model; a master unit (1), a slave unit (2), a PC (3), an endoscope monitor (4), a robot operation monitor (5), the colonoscope (Olympus PCF-240; Tokyo, Japan) (6), an endoscope system (Olympus CLV-U240D, CV-240; Tokyo, Japan) (7) and the colonoscopy training model (8). **B** The master unit of EOR version 4 consists of a knob-like rotating part (1) (rotating knob), a joystick (2), and a linear slider (3).

The EOR system has evolved progressively from version 1^12^, which was controlled via a joystick. In version 3, a proprietary master unit with haptic feedback was introduced, enabling intuitive one-handed insertion, retraction, and rotation operations while providing force sensation^13,14^. Building on this, version 4 incorporated haptic feedback into all operations, including vertical and horizontal angulation of the scope tip (Japanese Patent No. 7401075) (Fig. 1, Supplementary Video 1). EOR ver.4 is a system capable of complete monitoring of 16 parameters, including force sensation, angle, speed, and scope insertion length for all operations, recorded at a minimum resolution of 1/1000 second, together with synchronized endoscopic video image data recorded in high definition, and both data streams can be annotated. In this study, a single expert endoscopist used EOR ver.4 to perform total colonoscope insertion using Insertion Pattern 1 of a colonoscope training model (Kyoto Kagaku Co., Ltd.), generating a teaching dataset from 100 insertions recorded at 1/100-second intervals. Of these, 12 were used to develop AI models. The expert endoscopist had experience with over 10,000 colonoscopy procedures.

### Development of an AI model for autonomous insertion and its mechanism of operation

#### Selection and training of the AI models used to construct a proprietary AI model for autonomous insertion

To construct a proprietary AI model capable of autonomous insertion, the existing AI models YOLOv5^15^ and DenseNet-121 were used^16^. The former is an object detection model, which was used to predict the direction of angulation. The latter is a convolutional neural network (CNN) model for image recognition, which was used to predict the state of advancement (forward), retraction (backward), and angulation speed.

**Fig. 2:**
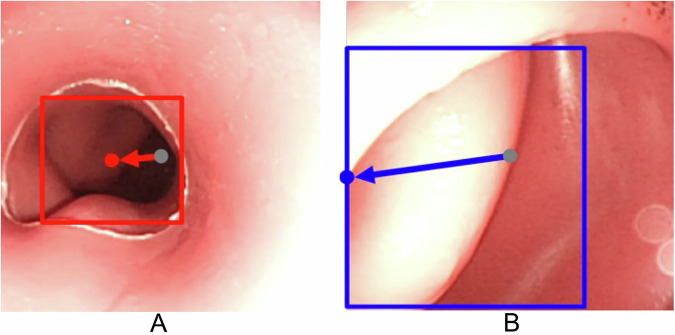
Angulation direction based on YOLOv5. **A** Hole: The detected Hole is enclosed by the bounding box (red frame). The angulation direction (red arrow) is determined as being from the center coordinates of the display (gray dot) to the center coordinates of the bounding box (red dot). **B** Fold: When the Fold class protruding from the right side is detected, to avoid the fold, the angulation direction (blue arrow) is determined as being toward the center coordinates (blue dot) of the left edge of the bounding box (blue frame).

The training dataset for YOLOv5 was created by annotating images extracted from the endoscopic video image data in the teaching dataset. Specifically, annotations were made by enclosing the target regions towards which the endoscope was to be angled within the endoscopic images. These enclosed regions are referred to as bounding boxes, and they are categorized into seven classes according to state of advancement on the image: “Hole,” “Gap,” “Wall,” “Fold (Right),” “Fold (Left),” “Fold (Up),” and “Fold (Down).” “Hole” refers to regions in which the colonic lumen is visible. “Gap” refers to regions in which the elongated, flattened lumen is visible. “Wall” refers to regions in which the intestinal wall is visible throughout the entire endoscopic image. For these three classes, the angulation direction was determined using the coordinates at the center of the bounding box. Specifically, the coordinates from the center of the image to the center of the bounding box defined the direction of angulation (Fig. 2A). “Fold” refers primarily to regions in which colonic folds, such as those in the sigmoid colon, are prominently visible. In the sigmoid colon, folds overlap, resulting in complex structures. For this reason, images of colonic folds, such as those in the sigmoid colon, were annotated as a separate class from other image types. Folds were categorized into four classes based on the direction from which they projected: “Right,” “Left,” “Up,” and “Down.” For example, in the case of a fold projecting from the right (Fold [Right]), the direction of angulation was determined using the coordinates at the center of the left edge of the bounding box. Specifically, the coordinates from the center of the image to the center of the bounding box edge defined the direction of angulation (Fig. 2B). For these four classes, the angulation direction was determined using the center of the bounding box edge opposite the direction in which the fold projects.

The training dataset for DenseNet-121 was obtained using endoscopic images from the teaching dataset and their corresponding manipulation data. Five types of manipulation data were used: insertion length of the endoscope [mm]; torque applied during left–right angulation [N·m]; torque applied during up–down angulation [N·m]; torque applied during scope rotation [N·m]; and reactive force in the insertion direction during advancement [N]. For each paired set of images and manipulation data, the scope manipulation speed was calculated, and a one-hot vector label corresponding to the speed classification was assigned, thereby generating the final training dataset. For forward and backward manipulation, speeds were labeled in three categories: “Forward” for 15 mm/s and above; “Back” for –15 mm/s and below; and “Stop” for all other speeds. For angulation, speeds were labeled in three categories: “Slow” for ≤25 mm/s; “Normal” for 25 mm/s to <62.5 mm/s; and “Fast” for ≥62.5 mm/s.

Training for the above two models was primarily conducted using PyTorch, a machine learning library for Python.

**Fig. 3:**
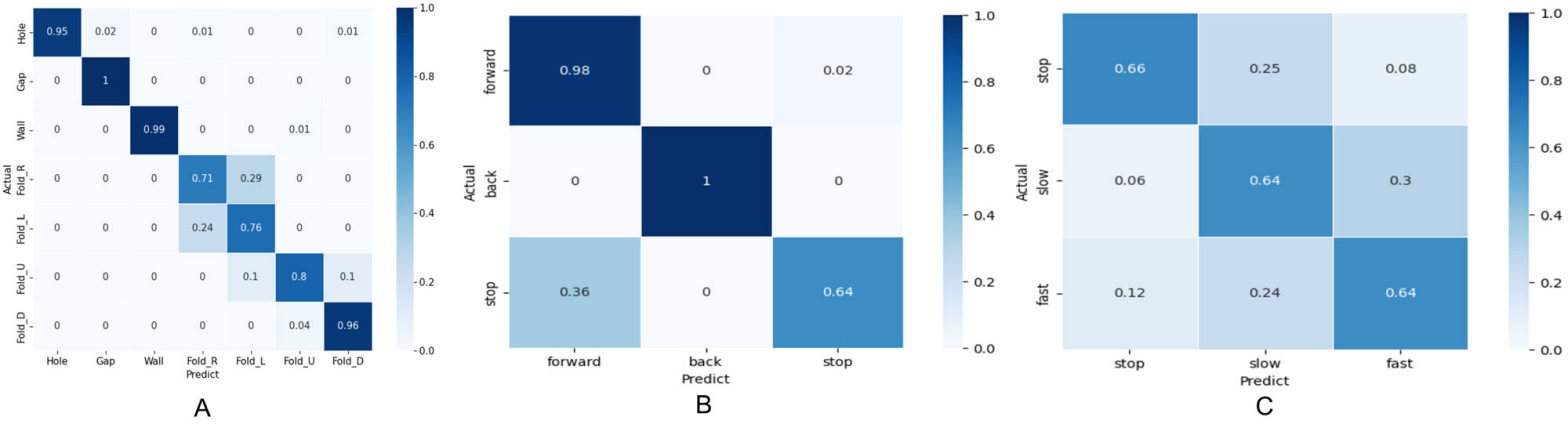
Confusion matrices from learning by YOLOv5 and DenseNet-121 training results. **A** YOLOv5 confusion matrix. **B** DenseNet-121 confusion matrix for forward/backward manipulation. **C** DenseNet-121 confusion matrix for angulation. (vertical axis, actual = actual measurements; horizontal axis: predict = predictions).

For YOLOv5, the training dataset consisted of 2100 images, the test dataset consisted of 470 images, and training was performed over 1000 epochs with a batch size of 16. The confusion matrix resulting from this training process is shown in Fig. 3A. The classification accuracy exceeded 95%, except for the Fold class, where the accuracy was 74% to 96%. It was considered that the situation in the direction of scope advancement could be predicted using the model weights obtained from the results of this training. Misclassifications were more frequent in the Fold class than in other classes, particularly between the Fold subclasses, but it was decided to adopt the weights obtained from this training in light of the fact that even manual insertions are conducted while continuously correcting decisions.

The training data for DenseNet-121 included 1745 training images and 300 test images for forward/backward manipulation, together with 1443 training images and 187 test images for angulation. To ensure a sufficient number of training images, several image transformations were randomly combined and applied to the model input data. (The optimization method used was Adam^17^, with a learning rate set to 0.0005. Mean squared error was used as the loss function for both forward/backward manipulation and angulation. After calculating the individual losses, a weighted sum was calculated for each batch, with the loss for forward/backward manipulation weighted at 0.7, and the loss for angulation weighted at 0.3.) Training was conducted over 5000 epochs with a batch size of 128. The resulting confusion matrices for forward/backward manipulation and angulation are shown in Fig. 3B and Fig. 3C, respectively. The average per-batch classification accuracy was 95.3% for forward/backward manipulation and 66.9% for angulation. An analysis of the classification accuracy for each class showed particularly high accuracy for the “Forward” and “Back” classes in forward/backward manipulation, whereas accuracy for the remaining classes was only around 60%. However, the weights obtained from this training were adopted for use in the system given that correct predictions were achieved for the majority of the data, and that “Forward,” which accounts for the majority of endoscope insertion maneuvers, had especially high accuracy.

Based on the above, a proprietary AI model for autonomous insertion was successfully developed using datasets generated for YOLOv5 and DenseNet-121.

#### Mechanism of operation using the AI model for autonomous insertion

Two personal computers (PCs) were used: one PC ran the program for EOR ver.4 (EOR ver.4 PC), and the other ran the program for the AI model (AI PC). These two PCs communicated across the network via inter-process communication using named pipes.

First, the EOR ver.4 PC sends a message to the AI PC to predict the appropriate operation. Upon receiving the message, the AI PC acquires the endoscopic image output from the flexible endoscope system and uses it to predict the appropriate endoscope operation. Finally, the AI PC sends the result of its prediction back to the EOR ver.4 PC, which then operates the EOR Ver.4 system accordingly based on the received instruction. The above process, including communication and AI prediction, was executed within 0.05 seconds, and by repeating this cycle every 0.05 seconds, autonomous operation was achieved. The forward speed was set to 6.5 mm/s, the backward speed to 4.0 mm/s, and the angulation speed was determined based on the prediction by the AI. For safety, the upper limit of force applied in the forward/backward direction was set to 20 N, and the torque limits for angulation in all directions were set to 0.5 N·m.

With these specifications, ACRS was completed.

### Study design and protocol

**Fig. 4:**
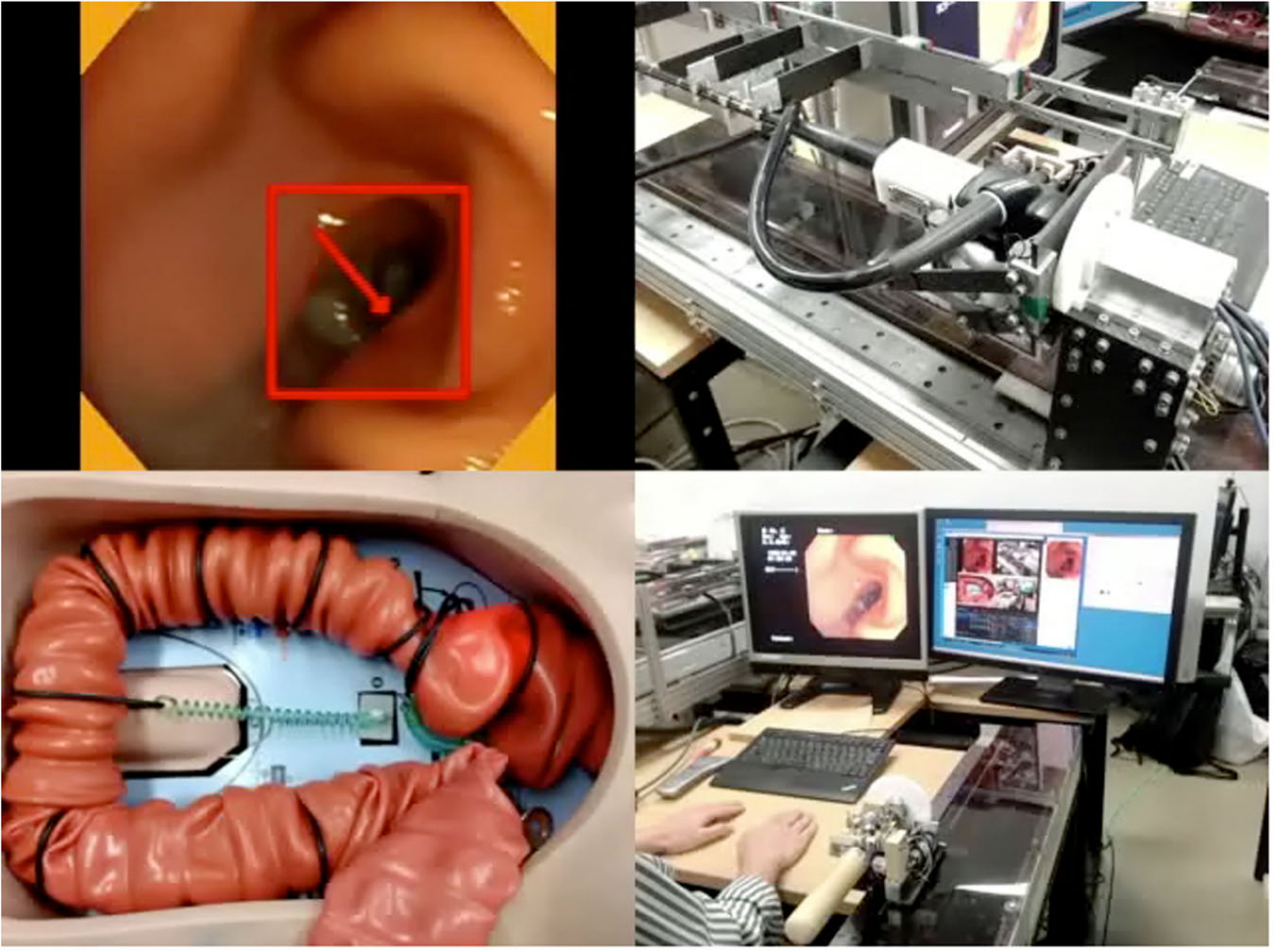
The robot operation monitor that displays four divided images. These show the operation, as captured by the three robot operation cameras, of the master unit (right lower), slave unit (right upper), colonoscopy training model (left lower), and the endoscope monitor (left upper).

**Fig. 5:**
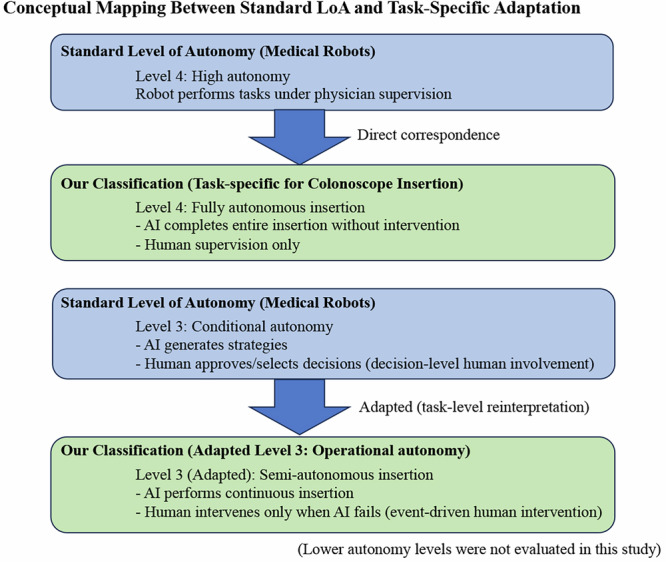
Conceptual mapping between the standard Level of Autonomy classification and our task-specific adaptation for autonomous colonoscope insertion.　Standard Level 4 autonomy directly corresponds to fully autonomous colonoscope insertion in this study. Standard Level 3 conditional autonomy was operationally adapted as semi-autonomous insertion, in which the AI performs continuous insertion but human intervention is required when the AI fails to determine an appropriate action. Lower autonomy levels were not evaluated in this study.

Automated insertion was performed using the ACRS. The entire automated insertion process was recorded along with time measurements using a four-panel video configuration of the endoscope screen, master unit, slave unit, and colonoscope training model (Fig. 4, Supplementary Video 2). The Level of Autonomy classification (LoA) for medical robots defines Level 3 (conditional autonomy) as systems in which the robot generates task strategies but requires human approval or selection of decisions, and Level 4 (high autonomy) as systems capable of performing tasks under physician supervision^18^ In this study, trials in which completely automated insertion was successfully achieved were designated Level 4. Trials that were completed but required manual intervention to pass through the location where the AI failed to make a decision were classified as Level 3. In contrast to the original definition focusing on decision-level supervision, we defined Level 3 operationally as trials in which the AI system required human intervention to overcome situations where it failed to determine an appropriate action during continuous insertion (Fig. 5).

### AI insertion performance

The success rate for Level 4 insertions and the mean insertion time (minutes) were determined. In addition, the mean insertion time (minutes) for Level 4 trials was compared with that of manual insertions performed by the expert (100 insertions) and trainees. The trainees had experience performing fewer than 200 colonoscopy procedures, and they performed five consecutive insertion trials after completing two practice sessions to familiarize themselves with the operation of the EOR ver.4 master unit.

### Operational parameter analysis

Operational parameters of colonoscope manipulation were quantitatively analyzed using data recorded from the robotic system. The parameters included angulation (left, up, and down), rotational torque (clockwise and counterclockwise), and forward insertion force. Expert teaching data were obtained from a single highly experienced endoscopist who performed 100 colonoscopes insertions in the colon model. Among these, 12 representative procedures that were used for AI training were selected and included in the present analysis to ensure consistency between training data and evaluated expert performance.

Trainee data were collected from less experienced operators, and AI-driven insertion data were recorded during autonomous procedures. For each parameter, mean values were calculated across all insertions and compared among expert, trainee, and AI groups.

### Analysis of AI decision corrections

AI decision corrections were defined as backward movements initiated by the system when forward insertion was judged to be inappropriate. These events were identified by retrospective review of recorded procedure videos rather than automated logging. Each backward movement was manually counted and assigned to anatomical segments of the colon, including the sigmoid colon, splenic flexure, and hepatic flexure. The frequency of backward movements per insertion was calculated for each anatomical region.

### Manual intervention analysis

Manual intervention was defined as operator takeover when the AI system was unable to determine the next insertion maneuver. These events were identified through retrospective video review and manually recorded. The anatomical location of each intervention was categorized into predefined colon segments. The number and distribution of manual interventions were analyzed across Level 3 insertions.

### Statistical analysis

Data are expressed as mean ± standard deviation (SD) values. The normality of continuous variables was assessed for each group using the Kolmogorov–Smirnov test. Because normality was not confirmed in at least one group, non-parametric tests were used. The prespecified pairwise comparisons were AI versus Expert and AI versus Trainee. These comparisons were performed using the Mann–Whitney U test, and Bonferroni-adjusted *p* values were calculated by multiplying the unadjusted p values by two. A *p*-value of <0.05 was considered significant. For binomial proportions, including the Level 4 success rate, 95% confidence interval (Cl) were calculated using the Clopper-Pearson exact method. Measurements were obtained from independent samples. All analyses were performed using SPSS version 31.0.2.0 (IBM Corp., Armonk, New York, USA).

### Use of artificial intelligence tools

A large language model (ChatGPT, OpenAI) was used to assist with language editing and drafting of the manuscript. The model was not used for data analysis, interpretation of results, or generation of scientific conclusions. All content was critically reviewed, verified, and approved by the authors, who take full responsibility for the integrity and accuracy of the work.

### Ethics statement and training data source

This study was conducted using a standardized colonoscopy training model and did not involve human participants, patient images, patient data, or biological specimens. The endoscopic images used to train the AI model were not obtained from publicly available datasets. They were generated specifically for this study from insertions performed by an expert endoscopist using EOR ver.4 in Pattern 1 of the standardized colonoscopy training model. The training dataset consisted of endoscopic images synchronized with robotic operation parameters recorded during these model-based insertions.

Because all images and operation data were obtained from a non-biological training model, and because no human participants, patient-derived data, identifiable information, or animal specimens were used, specific institutional review board approval and informed consent were not required for this study. Therefore, considerations related to demographic representation were not applicable. The study design and reporting were conducted in accordance with principles of research integrity, transparency, and responsible use of artificial intelligence technologies.

## Results

### AI insertion performance

A total of 87 consecutive automated insertion trials were performed using the system described above. Of these, 15 trials were excluded because image interpretation was not possible due to gel-like lubricant adhering to the tip of the endoscope. All 72 remaining trials achieved Level 3 or higher. Level 4 was attained in 62 trials, with a mean insertion time of 2.92 ± 1.20 minutes; Level 3 was attained in 10 trials, with a mean insertion time of 5.22 ± 1.59 minutes. The Level 4 success rate was 86.1% (62/72; 95% Cl, 75.9–93.1%).

Manual insertion by one expert over 100 trials yielded a mean insertion time of 1.43 ± 0.32 minutes. Manual insertion by five trainees over 25 trials yielded a mean insertion time of 2.97 ± 1.32 minutes.

Although automated insertion at Level 4 took significantly longer than insertion by the expert (*p* < 0.001), it was comparable to that of the trainees (*p* = 1.0) (Table 1).

**Table 1:** Comparison of insertion time between AI (Level 4), experts, and trainees

	Time (mean ± SD), min	*P* value vs AI model: Level 4
AI model: Level 4 (*n* = 62)	2.92 ± 1.20	-
Experts (*n* = 100)	1.43 ± 0.32	1.26 × 10^−^^25^
Trainees (*n* = 25)	2.97 ± 1.32	1

Min; minutes, Significant at <0.05.

Data are presented as mean ± standard deviation (SD). Sample sizes were AI Level 4 (*n* = 62), AI Level 3 (*n* = 10), expert (*n* = 100), and trainees (*n* = 25). Comparisons were performed using the Mann–Whitney U test with Bonferroni correction. All tests were two-sided.

### Operational parameter analysis

Operational parameters of the AI-driven insertion were compared with expert teaching data and trainee performance (Tables 2 and 3). Angulation parameters of the AI model were generally comparable to those observed in expert teaching data, whereas trainees demonstrated significantly larger angulation movements. In particular, left and upward angulations were markedly greater in trainees than in the AI model (both *p* < 0.001).

**Table 2:** Comparison of angle parameters between AI, expert teaching data, and trainees

	Experts used as teaching data (*n* = 12) Mean ± SD	AI model: Level 4 (*n* = 62) Mean ± SD	Trainees (*n* = 25) Mean ± SD	*P*-value: AI model vs experts	*P*-value: AI model vs trainees
Right angle (degrees)	35.29 ± 12.53	37.50 ± 7.68	33.81 ± 12.20	1	0.044
Left angle (degrees)	24.47 ± 6.75	25.12 ± 8.05	46.29 ± 16.65	1	1.12 × 10^−7^
Up angle (degrees)	34.01 ± 6.71	28.34 ± 12.39	48.20 ± 13.21	0.023	1.12 × 10^−7^
Down angle (degrees)	30.35 ± 7.56	70.47 ± 12.79	29.86 ± 15.05	1.06 ×10^-7^	2.89 × 10^−11^
Clockwise rotation (degrees)	3.10 ± 2.77	0.18 ± 0.34	21.16 ± 18.55	3.37 ×10^-5^	1.17 × 10^−^^12^
Counter- clockwise rotation (degrees)	6.26 ± 2.47	0.89 ± 1.15	19.00 ± 33.60	2.77 ×10^-7^	1.46 × 10^−5^

Significant at <0.05.

Data are presented as mean ± standard deviation (SD). Sample sizes were AI (*n* = 65), expert teaching data (*n* = 12), and trainees (*n* = 25). Comparisons were performed using the Mann–Whitney U test with Bonferroni correction. All tests were two-sided.

**Table 3:** Comparison of force and torque parameters between AI, expert teaching data, and trainees

	Experts used as teaching data (*n* = 12) Mean ± SD	AI model: Level 4 (*n* = 62) Mean ± SD	Trainees (*n* = 25) Mean ± SD	*P*-value: AI model vs experts	*P*-value: AI model vs trainees
Right angle (N.m)	0.024 ± 0.010	0.023 ± 0.007	0.046 ± 0.020	0.596	4.07 × 10^−7^
Left angle (N.m)	0.040 ± 0.004	0.032 ± 0.007	0.040 ± 0.008	2.17 × 10^−5^	4.06 × 10^−7^
Up angle (N.m)	0.046 ± 0.012	0.055 ± 0.010	0.048 ± 0.012	0.008	5.67 × 10^−4^
Down angle (N.m)	0.041 ± 0.009	0.031 ± 0.010	0.054 ± 0.013	0.004	1.71 × 10^−10^
Clockwise rotation (N.m)	0.031 ± 0.014	0.003 ± 0.001	0.078 ± 0.037	9.78 × 10^−8^	7.23 × 10^−13^
Counter- clockwise rotation (N.m)	0.044 ± 0.019	0.004 ± 0.004	0.067 ± 0.052	1.36 × 10^−7^	1.10 × 10^−12^
Push (N)	5.54 ± 0.65	8.18 ± 0.82	6.07 ± 1.46	1.15 × 10^−7^	8.62 × 10^−8^
Pull (N)	0.73 ± 0.85	1.66 ± 1.05	2.41 ± 0.80	0.001	5.08 × 10^−4^

Significant at <0.05.

Data are presented as mean ± standard deviation (SD). Sample sizes were AI (*n* = 62), expert teaching data (*n* = 12), and trainees (*n* = 25). Comparisons were performed using the Mann–Whitney U test with Bonferroni correction. All tests were two-sided.

Force analysis revealed distinct manipulation patterns between the three groups. Rotational torque was minimal in the AI system compared with both experts and trainees (*p* < 0.001). The AI model applied slightly greater forward insertion force than expert teaching data (8.18 ± 0.82 N vs 5.54 ± 0.65 N, *p* < 0.001), while trainee forces were intermediate. These findings indicate that the AI model reproduced insertion maneuvers closer to expert behavior than to trainee manipulation patterns.

### AI decision correction

AI decision corrections involving backward movements were analyzed in Level 4 insertions (*n* = 62). Backward movements occurred predominantly in the sigmoid colon (3.77 ± 10.49 per insertion), whereas substantially fewer corrections were observed at the splenic flexure (0.58 ± 2.08) and hepatic flexure (0.52 ± 1.88), resulting in a total of 4.87 ± 10.97 corrections per insertion (Table 4).

**Table 4:** Frequency of AI decision corrections involving backward movements during Level 4 insertions

	Part of colon			
Frequency	Sigmoid colon	Splenic flexure	Hepatic flexure	Total
Mean ± SD (range)	3.77 ± 10.49 (0-77)	0.58 ± 2.08 (0–12)	0.52 ± 1.88 (0–12)	4.87 ± 10.97 (0–77)

Data are presented as mean ± standard deviation (SD) and range. The number of backward movements was analyzed in Level 4 insertions (*n* = 62) across different colonic segments. Backward movements occurred most frequently in the sigmoid colon, with fewer corrections observed at the splenic and hepatic flexures. Measurements represent the number of correction events per insertion.

### Manual intervention

Manual intervention was required in Level 3 insertions (*n* = 10) when the AI system was unable to determine the next insertion maneuver. Most interventions occurred in the sigmoid colon (7 cases), while fewer were required at the hepatic flexure (2 cases) and splenic flexure (1 case). Only one manual intervention was performed per insertion (Table 5).

**Table. 5:** Frequency and distribution of manual interventions during Level 3 insertions

	Part of colon		
Frequency	Sigmoid colon	Splenic flexure	Hepatic flexure
	7	1	2

Data represent the number of insertions requiring manual intervention at each colonic segment during Level 3 insertions (*n* = 10). Manual intervention was required when the AI system failed to determine the next insertion maneuver. Only one intervention was performed per insertion. Most interventions occurred in the sigmoid colon, with fewer interventions at the hepatic and splenic flexures.

## Discussion

The following two principles guided the development of this system. First, when pursuing AI-driven autonomy, it is essential that the current level of care in colonoscopy be maintained, and this is the ultimate goal of the system’s development. Second, although the main focus of medical AI is currently diagnostic imaging^19,20^, physicians remain the principal agents of action in technical or therapeutic procedures, and the focus of development has therefore been on supporting practitioners, such as through 3D reconstruction of surgical organs or navigational guidance within the surgical site^21,22^. In considering medical procedures or treatments currently performed by the physician as the main agent that could be handled by an AI-driven autonomous robotic system, we focused on total colonoscopy as a procedure that demands a high level of skill but operates within limited control axes and degrees of freedom, making it a technically achievable target for development.

Current colonoscopy practice relies on both the colonoscope itself, which is designed to enable highly precise manipulation, and the skill of the endoscopist who operates it^10,11^. The establishment of axis-keeping shortening (AKS) and other techniques, insertion in patients with a history of abdominal surgery or complex bowel anatomy^11^, high adenoma detection rates (ADRs) through detailed observation^23^, and procedures such as endoscopic submucosal dissection (ESD)^24,25^ for early-stage colorectal cancer would not be achievable without both of these elements. An attempt to achieve autonomous colonoscope insertion using AI has been reported by Hwang et al^26^. However, the platform used to mount the colonoscope had a similar configuration to the first-generation EOR system. In other words, because it lacked the haptic feedback functionality essential for precise manipulation, this system was limited to simple training models for the bowel and could not be applied to more complex situations. A platform that provides haptic feedback across all aspects of colonoscope manipulation (insertion and retraction, rotation, and angulation of the scope tip in all directions) and enables comprehensive monitoring of both manipulation and corresponding image data is a requirement for AI-driven autonomous insertion. To meet this requirement, EOR ver.4 was developed. However, several challenges were identified in the results of these autonomous insertion trials.

Colonoscope insertion remains the most technically challenging phase of colonoscopy. By focusing specifically on insertion control, the present work addresses a fundamental bottleneck in the development of autonomous colonoscopy systems. Observation and lesion detection tasks, which are comparatively structured visual recognition problems, may be integrated into the system in subsequent stages after stable insertion control has been established. Our primary objective in the current study was to determine whether the AI could reliably learn the fundamental behaviors required for successful cecal intubation under controlled conditions. Therefore, we used Pattern 1 of the Kyoto Kagaku colonoscopy model as a first feasibility step. Pattern 1 was chosen deliberately to focus on whether the system can learn the critical maneuvers for reaching the cecum without the added complexity of looping or torsion. Moreover, an important aspect of the present study is the use of insertion data from a single highly experienced endoscopist for the initial training of the AI model. This approach was intentionally adopted to allow the extraction of clearly interpretable expert insertion maneuvers under controlled anatomical conditions. In early-stage development of autonomous robotic systems, the inclusion of heterogeneous motion patterns from multiple experts may introduce variability that can obscure the core strategies underlying successful insertion^27,28^. By focusing on carefully curated expert motion data, the present model was able to learn stable insertion behaviors while avoiding potentially misleading or inconsistent motion patterns. In future studies involving more complex colon configurations (Patterns 2–6, which include redundant sigmoid anatomy and loop formation), training datasets from multiple experienced endoscopists will be incorporated to enable the AI system to learn strategies for loop formation, loop reduction, and operator variability.

In Pattern 1, the expert operator did not need to activate lens irrigation or suction, as insertion was performed without significant contamination. However, in our experiments, we observed cases in which lubricant accumulation obscured the endoscopic view (15 trials, as reported in the Results). In the current EOR ver.4 system, lens cleaning, suction, and air insufflation are operated manually via foot-switch controls. Importantly, these functions are already available at the hardware level, and their integration into the AI control framework is therefore technically feasible without major system modification. In future iterations, we plan to implement automated control of these functions. Specifically, the AI will be programmed to activate lens irrigation and suction when visual clarity is compromised, and to dynamically adjust insufflation—for example, reducing air supply when there is a risk of loop formation. Particularly, the ability to autonomously manage visual clarity and luminal distension will be critical for extending the system to more complex anatomical conditions. Incorporating these capabilities is expected to improve system robustness against common procedural challenges, including fluid contamination, debris, and variability in luminal distension.

Additional analyses of operational parameters provided further insight into the behavioral characteristics of the AI-driven insertion process. The angulation parameters of the AI model were generally comparable to those observed in the expert teaching data, whereas trainees tended to use significantly larger angulation movements and higher torques. These findings suggest that the AI system reproduced insertion maneuvers closer to expert behavior than to novice strategies. Interestingly, rotational manipulation was minimal in the AI system compared with both experts and trainees, indicating that the AI relied primarily on controlled angulation and forward advancement rather than excessive shaft rotation. Although the AI applied slightly greater forward insertion force than the expert teaching data, these forces remained within the predefined safety limits of the robotic system.

The analysis of AI decision corrections provides additional insight into how the system responds to challenging anatomical situations. Most backward movements occurred in the sigmoid colon, while substantially fewer corrections were observed at the splenic and hepatic flexures. This distribution is consistent with clinical experience, as the sigmoid colon is widely recognized as the most technically demanding segment during colonoscope insertion due to loop formation and anatomical variability^29^. Similarly, manual interventions in Level 3 cases were also concentrated in the sigmoid colon. These observations suggest that the AI system may already capture aspects of clinically relevant insertion strategies when encountering difficult anatomical regions.

An important feature of the present study is the stepwise translational development strategy underlying the ACRS platform. The current work represents the initial phase of this pathway, focusing on the feasibility of autonomous insertion in a controlled colon model. Future studies will extend the system to more complex colon configurations and incorporate training data from multiple endoscopists to enable the AI to learn strategies for loop formation and reduction. Subsequent validation in ex vivo porcine colon models followed by in vivo animal experiments will further evaluate the safety and robustness of the system before exploratory clinical studies. Such a staged development framework may facilitate the safe translation of autonomous endoscopic robotics into clinical practice.

While the present study focuses on autonomous colonoscope insertion, complete colonoscopy involves additional steps, including mucosal inspection, lesion detection, and therapeutic intervention. Following the establishment of reliable insertion across broader anatomical conditions, the system will be extended to controlled withdrawal with systematic mucosal inspection by integrating the EOR platform’s mechanical capabilities with vision-based AI. Therapeutic functions may subsequently be incorporated. In a potential clinical workflow, the operator would supervise autonomous insertion and assume a more active role during withdrawal to validate findings and perform interventions. These functions will be developed in a stepwise manner after robust insertion has been achieved.

The ACRS incorporates multiple safety layers designed to ensure safe operation, including force-controlled actuation, an insertion force limit of ±20 N, an angulation torque limit of 0.5 Nm, and speed control across all operational axes. Furthermore, the system allows real-time manual override, is equipped with an emergency stop mechanism, and enables rapid conversion to conventional manual colonoscopy when necessary.

As the field of AI-assisted endoscopy continues to evolve, it is increasingly important that future development efforts consider translational actors in parallel with technical innovation. The successful clinical deployment of AI-assisted endoscopy has underscored that improvements in technical performance alone do not necessarily translate into clinical impact. Large prospective trials have demonstrated enhanced detection outcomes with computer-aided systems^30^; however, real-world implementation studies highlight the critical roles of workflow compatibility, clinician trust, and appropriate human oversight^31^. Human factors research further indicates that the safe integration of automation in high-stakes clinical environments depends on careful calibration of autonomy and supervision^32,33^. Accordingly, future development of autonomous insertion systems should incorporate adaptive supervision architectures together with rigorous, staged safety validation frameworks^18,34^.

It is especially important to emphasize that our study should be interpreted as an engineering proof of concept, not as a complete clinical application validation study. The validation was performed using Pattern 1 of a standardized colonoscopy training model, which does not reproduce the full anatomical and physiological complexity of clinical colonoscopy. In particular, this model does not adequately simulate redundant sigmoid anatomy, loop formation and reduction, torsion, peristalsis, patient movement, variable insufflation, luminal contamination, or in vivo tissue–scope interaction. Moreover, insertion in this configuration requires only limited colonoscope-specific maneuvering compared with difficult clinical cases. Therefore, the present findings demonstrate that the ACRS can acquire and execute basic autonomous insertion behavior under simplified conditions, but they do not establish readiness for clinical translation. Further validation will require a stepwise developmental pathway, including testing in more complex colonoscopy model configurations, especially those involving sigmoid loop formation, followed by ex vivo porcine colon experiments, in vivo animal studies, and carefully supervised exploratory clinical studies in selected low-risk cases.

In conclusion, this study demonstrates the proof-of-concept feasibility of autonomous colonoscope insertion using an AI-based robotic platform trained on expert operational data. Under simplified and highly controlled model conditions, ACRS achieved a high rate of fully automated insertion and showed insertion times comparable to those of trainees. However, the present findings should not be interpreted as evidence of clinical readiness, because the current validation did not reproduce the anatomical and physiological complexity of human colonoscopy, including redundant sigmoid anatomy, loop formation, luminal contamination, and in vivo tissue–scope interaction. Further stepwise validation in more complex colon models, ex vivo and in vivo animal experiments, and carefully supervised exploratory clinical studies will be required before translational or clinical application can be considered.

## Supplementary information

Transparent Peer Review file

Description of Additional Supplementary Files

Supplementary Video 1

Supplementary Video 2

## Data Availability

The source data supporting Tables 1–5 are available in figshare^35^ at 10.6084/m9.figshare.30244321. The training data used to develop the autonomous insertion model were generated in this study from endoscopic images and synchronized robotic operation data obtained during insertions performed by an expert endoscopist using EOR ver.4 in a standardized colonoscopy training model. These training data are not publicly available because they include large video-based operational datasets and proprietary robotic-control data. Access to the training data may be provided by the corresponding author upon reasonable request, subject to technical feasibility. Requests should be addressed to Keiichiro Kume, MD, PhD, at k-kume@med.uoeh-u.ac.jp. Supplementary Video 1: The EOR ver.4 was used to perform total colonoscopy using a colonoscopy training model with an insertion time to the cecum of 53 seconds (uncut video). Supplementary Video 2**:** The ACRS was used to perform total colonoscopy using a colonoscopy training model with an insertion time to the cecum of 152 seconds (un cut video).
