# Supplementary material for: In vitro development of the Autonomous Colonoscope Robot System (ACRS) for fully automated colonoscope insertion
Source: Commun Med (Lond). 2026 Jun 30;6:366. doi: 10.1038/s43856-026-01747-8 (PMC13319117; doi:10.1038/s43856-026-01747-8)
Supplement: Supplementary file 2 — Description of Additional Supplementary Files [file 43856_2026_1747_MOESM2_ESM.docx]

**Description of Additional Supplementary Files**

Supplementary Video 1: The EOR ver.4 was used to perform total colonoscopy using a colonoscopy training model with an insertion time to the cecum of 53 seconds (un cut video).

Supplementary Video 2: The ACRS was used to perform total colonoscopy using a colonoscopy training model with an insertion time to the cecum of 152 seconds (un cut video)
