# Supplementary material for: In vitro development of the Autonomous Colonoscope Robot System (ACRS) for fully automated colonoscope insertion
Source: Commun Med (Lond). 2026 Jun 30;6:366. doi: 10.1038/s43856-026-01747-8 (PMC13319117; doi:10.1038/s43856-026-01747-8)
Supplement: Supplementary file 1 — Transparent Peer Review file [file 43856_2026_1747_MOESM1_ESM.pdf]

nature portfolio

Peer Review File

In vitro development of the Autonomous Colonoscope Robot System (ACRS) for fully automated colonoscope insertion

Corresponding Author: Dr Keiichiro Kume

This file contains all reviewer reports in order by version, followed by all author rebuttals in order by version.

Version 0:

Reviewer comments:

Reviewer #1

(Remarks to the Author)
Summary

This paper describes the development and testing of an autonomous robotic system for colonoscopy. The authors have built an AI driven robot using a master slave endoscopic platform with full haptic feedback.

They trained the system on insertion data from an expert endoscopist. The AI combined image recognition and motion prediction to control insertion, withdrawal, rotation and tip angulation without human input. All testing was performed in a simulated environment rather than in patients.
The study shows that fully autonomous colonoscope insertion is technically feasible and can reach trainee level performance. It highlights potential future applications for workforce support and training while acknowledging that further development and clinical validation are required before
real world use.

Key results
The work demonstrates that fully autonomous colonoscope insertion is technically feasible using an AI driven robotic platform without human intervention. The system achieved a high success rate (86% fully automated insertion). Insertion performance was slower than an expert endoscopist but closely matched that of trainee endoscopists showing consistent and reproducible behaviour. A key strength is the integration of real time analysis with force sensing and motion control allowing the robot to manage insertion, withdrawal, rotation and tip angulation. The AI was trained on high resolution procedure data from an expert with extensive clinical experience strengthening the plausibility of the model. Importantly the system operates using a standard clinical colonoscope rather than a
bespoke device-helping to support future translation to practice. These initial results suggest a credible foundation for the future development towards clinical application training support and workforce augmentation.

Validity
Whilst the manuscript does not contain flaws prohibiting publication, it does have important limitations that restrict interpretation and generalisability.

All experiments were performed in a colonoscopy training model rather than in patients-limiting validity as the system was not exposed to variable anatomy, peristalsis, looping or patient discomfort. Performance in a simulated model can’t be assumed to translate to clinical practice.
The AI was trained on data from a single expert endoscopist. This creates a risk of overfitting to one operator’s technique and limits robustness across different insertion styles.

The definition of success focusses on completion of insertion rather than clinically relevant outcomes. There is no assessment of mucosal visualisation, loop management or downstream diagnostic performance. I feel these omissions matter for clinical relevance.

Insertion times are used as a primary comparator, time alone is an incomplete quality metric (and doesn’t capture procedural safety or effectiveness). Just because performance matches trainees does not necessarily imply acceptable clinical quality.

Trials with poor image quality were excluded introducing selection bias and highlights a key failure mode that would be common in real world practice.

Despite these limitations, the methodology is consistent, results are transparently reported and the conclusions are largely cautious. The flaws do not prohibit publication but they do require clear framing as proof of feasibility rather than readiness for clinical use currently.

Originality
The conclusions are largely original within the current literature. Previous robotic colonoscopy systems have focussed on alternative propulsion mechanisms and earlier autonomous approaches using conventional scopes have been preliminary, lacked full haptic feedback or demonstrated only partial autonomy in simplified models.

This works shows fully autonomous insertion using a standard clinical colonoscope mounted on a robotic platform, trained on expert procedural data representing a meaningful advance.

Significance lies in demonstrating trainee level autonomous performance using vision based AI integrated with motion control and force control. This shifts the discussion from theoretical use to practical feasibility. The conclusions do not claim clinical readiness, they establish the proof of concept that extends beyond prior work.

The results are of immediate interest to several groups. Gastroenterologists and endoscopy service leads will recognise the relevance to workforce constraints, training and standardisation of insertion technique. Within Gastroenterology, the appeal will be more academic than clinical at present. Health systems researchers may find the interest in automation growing and perhaps help address quality variation and capacity.

Data and methodology
The data and methodology are reported with sufficient detail. The data are well characterised and the presentation is clear. The methodological approach is appropriate for a feasibility study. The authors clearly describe the robotic platform, AI system and control loop that enables the automatic insertion. The data quality is high.

However, the reliance on a single expert does limit the diversity in technique and reduces robustness. The dataset size for AI training is modest, especially given the complexity of the task.

The experimental validation is clearly defined using established autonomy levels and outcome measures are transparently reported. Comparisons with expert and trainee insertion times provide useful benchmarks.

Exclusion of trial due to image contamination reveals a key limitation of the data and the system's resilience to real world conditions.

Supplementary videos do enhance the transparency and allow readers to assess model performance and failure modes.

Appropriateness of statistics
The use of statistics is appropriate for the scope and aims of the study and transparently reported. The authors recognise that insertion time data don't follow a normal distribution and hence they use a non parametric test.

Mann Whitney U test with Bonferroni correction is suitable for comparing automated insertion times with expert and trainee performance. Probability values are accurately reported and interpreted correctly. Error reporting is generally clear and where variability is reported in text and tables, the metrics used are consistent.

Uncertainty is handled adequately. The analysis focuses almost entirely on insertion time as a single outcome measure. There is no formal qualification of uncertainty around success rates beyond simple proportions and no confidence intervals are provided. Whilst this isn't mandatory for a feasibility study the inclusion of Cis would strengthen the interpretation.

Conclusions
Data interpretation is cautious and largely valid. The comparison with expert and trainee insertion times is used to contextualise performance rather than to imply equivalence with expert practice. The authors acknowledge key limitations (longer insertion times, restricted training data and failure models related to image interpretation). Reliability is confined to a simulated environment and a single insertion pattern which limits external validity.

The conclusions are generally robust and aligned with the data presented. They are consistent and reliable within their state scope. The authors appropriately frame the work as a feasibility study and avoid claiming clinical readiness.

Suggested improvements
Several additions would substantially strengthen the work in a revision.

Testing across multiple colonoscopy training models with different anatomical configurations for looping and difficulty levels would help improve robustness and demonstrate adaptability beyond a single insertion pattern.

Incorporating training data from multiple expert endoscopists would reduce operator specific bias and improve

generalisability.

Prospective testing in ex vivo/ animal models would provide an important intermediate step towards clinical relevance. This would allow assessment of safety surrogates such as applied forces, loop creation and recovery from loss of luminal view in a dynamic setting. Withdrawal of the scope and demonstrations of lesion detection/visualisation behind folds would also testing for relevance.

Expanded outcome measures would strengthen interpretation-these could include force profiles, frequency of backward movements, number of AI decision corrections and time spent with no visible lumen rather than relying primarily on insertion time.

Analysis of failure modes would add value, reporting where and why Level 3 autonomy occurred and how the system recovered or failed would also help the reader understand the system limitations.

Improving robustness to common real-world issues eg lens contamination, suction use and variable insufflation would address some of the limitations.

References
The references are relevant and early work on the Endoscopic Operation Robot platform are correctly cited. There are a few areas where referencing could be strengthened. Inclusion of more recent clinical studies on AI assisted endoscopy adoption particularly those addressing clinician acceptance, workflow integration and safety would better contextualise translational relevance.

References on human factors, trust in automation and operator oversight in medical robotics would strengthen the manuscript and support discussion around autonomy and acceptance.

Clarity and context
The abstract is clear, structured and accessible to a broad readership. It succinctly states the clinical problem, describes the robotic and AI approach and reports the key outcomes in appropriate detail.
The rationale for focussing on autonomous insertion using a standard colonoscope is discussed and convincingly argued. The transition from clinical need to technical solution is coherent. The conclusions are appropriate and consistent with the abstract and results.

Overall, the manuscript communicates the aim and findings clearly, places the work in an appropriate clinical and technological context.

Inflammatory material
The manuscript doesn't contain language that is inappropriate or inflammatory. The language is appropriate for this journal.

Reviewer #2

(Remarks to the Author)
The manuscript by Kume et al entitled "Development of an autonomous robot for fully automated colonoscope insertion: The autonomous colonoscope robot system" stands in line with many other previous papers on robotic systems designed for colonoscopy. The concept of an autonomous colonoscope is older than 20 years, and authors themeselves published their first concept almost 15 years ago from now. Several systems including those that even became a clinical product are no longer available. Reasons are manifold but most likely are related to the many problems that occur when real patients and not simulators have been scoped.

New, and interesting in the present manuscript is the integration of an AI that enables fully automated insertion and scope passage. The paper is well written with a sound methodology. Nevertheless, I have some comments to make.

1. Insertion of the endoscope and passage of the scope to the caecum is less than half of the procedure. Detailed inspection is most relevant and endoscopic therapy should be performed once a lesion is detected. What is the concept of the authors to include these steps into their platform? Any suggestions for a clinical workflow?

2. The main limitation in routine colonoscopy is creation of loops that harm the patient. The simulater as used is a rather simple one, where creation of loops is hardly possible. I'd strongly suggest to further evaluate this topic and perform additional experiment in other simulators that measure forces and pressure during loop creation (e.g. the Mikoto simulator).

3. The Levels as used should be further explained. Without reading the cited reference, it is unclear what Level 3 and 4 stand for.

Reviewer #3

(Remarks to the Author)
I co-reviewed this manuscript with one of the reviewers who provided the listed reports. This is part of the Communications

Medicine initiative to facilitate training in peer review and to provide appropriate recognition for Early Career Researchers who co-review manuscripts.

Reviewer #4

(Remarks to the Author)
In this manuscript, the authors described about the study using an autonomous colonoscope insertion system based on AI-driven robotic control. The underlying concept and the technical sophistication of the developed platform are interesting and, from an engineering perspective, commendable.

However, in the current form, the study demonstrates technical feasibility only in a highly simplified setting of artificial colon model. Based on the provided videos, the experimental setup appears to require only minimal and straightforward maneuvers, and it remains unclear whether the proposed system would be capable of handling the anatomical complexity and variability encountered in real clinical colonoscopy.

In addition, the comparison of insertion time between the robotic system and human operators is problematic, as the operators were required to use a robotic interface (lever-based insertion and joystick-based angulation) that is fundamentally different from standard manual colonoscopy. This makes the comparison clinically unnatural and of limited interpretability.

Furthermore, the manuscript provides insufficient evaluation of safety and does not adequately address translational or clinical considerations. Overall, the work remains at the stage of a robotic proof-of-concept rather than a clinically oriented study.

- Major comments

1. Lack of clinical perspective and translational relevance
The present study does not include any form of clinical evaluation and remains close to an engineering proof-of-concept. From the perspective of translational or clinical research, which is a core focus of "Communication Medicine", there appears to be a fundamental mismatch between the study design and the journal’s scope. The clinical relevance and potential impact on patient care are not sufficiently demonstrated.

2. Oversimplified colon model and lack of realistic insertion difficulty
The Kyoto Kagaku colonoscopy training model (Pattern 1) used in this study does not adequately reproduce the most challenging part of colonoscope insertion, namely the tortuosity and looping tendency of the sigmoid colon. As shown in the provided videos, the insertion can be completed with only minimal angulation and forward advancement.
Experiments conducted under such conditions essentially demonstrate the ability of a robot to perform simple forward and backward movements, rather than clinically meaningful colonoscope navigation. At a minimum, validation should be performed using the more difficult model configurations (Patterns 2–5), which represent a longer and more redundant sigmoid colon with a higher propensity for loop formation.

3. Absence of shaft rotation and unrealistic manipulation patterns
According to the videos, neither human-operated nor AI-driven insertions involve substantial axial rotation of the colonoscope shaft. Instead, navigation is achieved almost exclusively through unidirectional tip angulation (predominantly upward), reflecting an overly idealized anatomical setting.
Such cases are extremely rare in clinical practice. Given that a standard commercial colonoscope was used in the model, it is unlikely that insertion without frequent shaft rotation would be feasible in real patients. Therefore, the current experimental setting lacks translational realism, and the reported success rate would likely be markedly lower in actual clinical environments.

4. Insufficient safety evaluation
The artificial colon model is made of non-biological resin materials, whose mechanical properties and pressure tolerance differ substantially from those of living tissue. Consequently, the current study does not allow for meaningful assessment of procedural safety, including risks of excessive wall stress, perforation, or mucosal injury. At a minimum, preclinical validation in animal models or a small-scale first-in-human safety study would be necessary to support any claims regarding clinical feasibility or safety.

Version 1:

Reviewer comments:

Reviewer #1

(Remarks to the Author)
the issues have been addressed in the rebuttal, I am happy with these changes

Reviewer #2

(Remarks to the Author)

Authors were able to adress all short comings of the previous version. I have no further commentsto be dealed with.

Reviewer #4

(Remarks to the Author)
As I noted in my previous review, the current study is still based on results obtained under a highly simplified experimental setting. At a minimum, further validation in animal models would strengthen the translational value of the work. Even if a training model is used, evaluation under conditions more closely resembling the human colon (especially the sigmoid colon) setting would be desirable.
The present setup appears to involve only limited colonoscope-specific maneuvering and may therefore be insufficient, even as an initial feasibility assessment.
In the revised version, no substantial additional validation has been provided, and I am not fully convinced that this concern has been adequately addressed. Overall, the work still seems to remain closer to an engineering proof-of-concept than to a translational research.

Open Access This Peer Review File is licensed under a Creative Commons Attribution 4.0 International License, which permits use, sharing, adaptation, distribution and reproduction in any medium or format, as long as you give appropriate credit to the original author(s) and the source, provide a link to the Creative Commons license, and indicate if changes were made.
In cases where reviewers are anonymous, credit should be given to 'Anonymous Referee' and the source.
The images or other third party material in this Peer Review File are included in the article's Creative Commons license, unless indicated otherwise in a credit line to the material. If material is not included in the article's Creative Commons license and your intended use is not permitted by statutory regulation or exceeds the permitted use, you will need to obtain permission directly from the copyright holder.
To view a copy of this license, visit https://creativecommons.org/licenses/by/4.0/

Point-by-Point Response to Reviewers (with tracked changes)

Dear Editor and Reviewers,
We sincerely thank the Editor and reviewers for their thoughtful evaluation and constructive comments. We have revised the manuscript extensively to improve clarity, methodological rigor, and translational relevance. In addition, minor typographical and editorial errors have been corrected throughout the manuscript.

All revisions are indicated in the marked-up manuscript. Below, we provide a detailed, point-by-point response, with explicit references to where changes have been made.

Reviewer 1

We are grateful for the reviewer's comprehensive and forward-looking suggestions, particularly regarding robustness, evaluation metrics, and translational development.

Comment1:
Testing across multiple colonoscopy training models…
Response:
We agree that validation under more complex anatomical conditions is important for demonstrating robustness. In this study, Pattern 1 was intentionally selected as a controlled first-step feasibility condition to isolate fundamental insertion behaviors. This rationale has now been clarified. We have also added a statement outlining future validation using more complex colon configurations (Patterns 2–6).
Track change:
Discussion
Added explanation of Pattern 1 as a first-step feasibility model
Colonoscope insertion remains the most technically challenging phase of colonoscopy. By focusing specifically on insertion control, the present work addresses a fundamental bottleneck in the development of autonomous colonoscopy systems. Observation and lesion detection tasks, which are comparatively structured visual recognition problems, may be integrated into the system in subsequent stages after stable insertion control has been established. Our primary objective in the current study was to determine whether the AI could reliably learn the fundamental behaviors required for successful cecal intubation under controlled conditions. Therefore, we used Pattern 1 of the Kyoto Kagaku colonoscopy model as a first feasibility step. Pattern 1 was chosen deliberately to focus on whether the system can learn the critical maneuvers for reaching the cecum without

the added complexity of looping or torsion. These descriptions were included in page 14 line 24- page 15 line 8.

Added future validation in complex colon configurations

An important feature of the present study is the stepwise translational development strategy underlying the ACRS platform. The current work represents the initial phase of this pathway, focusing on the feasibility of autonomous insertion in a controlled colon model. Future studies will extend the system to more complex colon configurations and incorporate training data from multiple endoscopists to enable the AI to learn strategies for loop formation and reduction. These descriptions were included in page 17 line 4- 12.

Comment 2:

Training data from multiple expert endoscopists…

Response:

We appreciate this important suggestion. In this initial study, we intentionally used data from a single highly experienced endoscopist to extract consistent and interpretable insertion strategies under controlled conditions. This rationale has now been clarified. We also added that future studies will incorporate datasets from multiple endoscopists to improve generalisability.

Track change:

Discussion

Added justification for single-expert dataset

Added future plan for multi-operator data integration

Moreover, an important aspect of the present study is the use of insertion data from a single highly experienced endoscopist for the initial training of the AI model. This approach was intentionally adopted to allow the extraction of clearly interpretable expert insertion maneuvers under controlled anatomical conditions. In early-stage development of autonomous robotic systems, the inclusion of heterogeneous motion patterns from multiple experts may introduce variability that can obscure the core strategies underlying successful insertion (Hu et al,2025, Mohammed et al, 2025). By focusing on carefully curated expert motion data, the present model was able to learn stable insertion behaviors while avoiding potentially misleading or inconsistent motion patterns. In future studies involving more complex colon configurations (Patterns 2–6, which include redundant sigmoid anatomy and loop formation), training datasets from multiple experienced endoscopists will be incorporated to enable the AI system to learn strategies for loop formation, loop reduction, and operator variability. These descriptions were included in page 15 line 9- 20.

Comment 3:
Ex vivo / animal validation…
Response:
We agree that preclinical validation is essential. We have added a staged translational development pathway, including validation in ex vivo porcine colon models followed by in vivo animal studies prior to clinical evaluation.
Track change:
Discussion
Added "stepwise translational development strategy" including ex vivo and in vivo validation
Subsequent validation in ex vivo porcine colon models followed by in vivo animal experiments will further evaluate the safety and robustness of the system before exploratory clinical studies. Such a staged development framework may facilitate the safe translation of autonomous endoscopic robotics into clinical practice. <u>These descriptions were included in page 17 line 9- 12.</u>

Comment 4:
Expanded outcome measures…
Response:
We agree that insertion time alone is a limited metric. Notably, the EOR system actually records multiple parameters synchronized with video. We added analyses of operational parameters (force, angulation, torque), AI decision corrections (backward movements).
Track change:
Methods
Added new subsections:
"Operational parameter analysis"
"AI decision correction"
Results
Added corresponding results (Tables 2–4 and related text)
Discussion
Added corresponding discussion
Additional analyses of operational parameters provided further insight into the behavioral characteristics of the AI-driven insertion process. The angulation parameters of the AI model were generally comparable to those observed in the expert teaching data, whereas trainees tended to use significantly larger angulation movements and higher torques.

These findings suggest that the AI system reproduced insertion maneuvers closer to expert behavior than to novice strategies. Interestingly, rotational manipulation was minimal in the AI system compared with both experts and trainees, indicating that the AI relied primarily on controlled angulation and forward advancement rather than excessive shaft rotation. Although the AI applied slightly greater forward insertion force than the expert teaching data, these forces remained within the predefined safety limits of the robotic system.

The analysis of AI decision corrections provides additional insight into how the system responds to challenging anatomical situations. Most backward movements occurred in the sigmoid colon, while substantially fewer corrections were observed at the splenic and hepatic flexures. This distribution is consistent with clinical experience, as the sigmoid colon is widely recognized as the most technically demanding segment during colonoscope insertion due to loop formation and anatomical variability (Kaminski, et al, 2017). <u>These descriptions were included in page 16 line 10- 25.</u>

Comment 5:

Failure mode analysis…

Response:

We have incorporated failure mode analysis through evaluation of Level 3 cases, including anatomical distribution of manual interventions and conditions in which the AI failed to determine appropriate actions.

Track change:

Methods

Added new subsections:

"Manual intervention" → *Added definition and evaluation approach*

Results

Added corresponding results (Tables5 and related text)

Discussion

Added corresponding discussion

Similarly, manual interventions in Level 3 cases were also concentrated in the sigmoid colon. These observations suggest that the AI system may already capture aspects of clinically relevant insertion strategies when encountering difficult anatomical regions. <u>These descriptions were included in page 16 line 25- page 17 line 3.</u>

Commet 6:

Robustness to real-world issues…

Response:
We have addressed this point by reporting cases of lens contamination (15 trials) and clarifying that irrigation, suction, and insufflation are already available at the hardware level. We further describe planned integration of these functions into the AI control framework.
Track change:
Discussion
Added explanation and future control strategy
In Pattern 1, the expert operator did not need to activate lens irrigation or suction, as insertion was performed without significant contamination. However, in our experiments, we observed cases in which lubricant accumulation obscured the endoscopic view (15 trials, as reported in the Results). In the current EOR ver.4 system, lens cleaning, suction, and air insufflation are operated manually via foot-switch controls. Importantly, these functions are already available at the hardware level, and their integration into the AI control framework is therefore technically feasible without major system modification. In future iterations, we plan to implement automated control of these functions. Specifically, the AI will be programmed to activate lens irrigation and suction when visual clarity is compromised, and to dynamically adjust insufflation—for example, reducing air supply when there is a risk of loop formation. Particularly, the ability to autonomously manage visual clarity and luminal distension will be critical for extending the system to more complex anatomical conditions. Incorporating these capabilities is expected to improve system robustness against common procedural challenges, including fluid contamination, debris, and variability in luminal distension. <u>These descriptions were included in page 15 line 21- page 16 line 9.</u>

Comment 7-8:
References and human factors…
Response:
We have strengthened the discussion by incorporating literature on AI adoption, workflow integration, clinician trust, and human factors in medical robotics.
Track change:
Discussion
Added human factors, trust in automation, and clinical integration references
As the field of AI-assisted endoscopy continues to evolve, it is increasingly important that future development efforts consider translational actor in parallel with technical innovation. The successful clinical deployment of AI-assisted endoscopy has underscored

that improvements in technical performance alone do not necessarily translate into clinical impact. Large prospective trials have demonstrated enhanced detection outcomes with computer-aided systems (Wang, et al, 2019); however, real-world implementation studies highlight the critical roles of workflow compatibility, clinician trust, and appropriate human oversight (Messmann, et al, 2022). Human factors research further indicates that the safe integration of automation in high-stakes clinical environments depends on careful calibration of autonomy and supervision (Lee, et al, 2004, Hoff, et ai, 2015). Accordingly, future development of autonomous insertion systems should incorporate adaptive supervision architectures together with rigorous, staged safety validation frameworks (Yang, et al, 2017, Char, et al, 2018). These descriptions were included in page 18 line 3-13.

Comment 9:

Confidence intervals…

Response:

We have added 95% confidence intervals for binomial proportions using the Clopper–Pearson exact method.

Track change:

Methods (Statistical analysis)

Added description of CI calculation

Results (AI insertion performance)

Added 95% CI for success rate

Reviewer 2

We thank the reviewer for highlighting important clinical and conceptual considerations.

Comment 1:

Integration of observation and therapy…

Response:

We agree that these components are essential for clinical application. We have clarified that this study focuses on insertion as a foundational control problem and added that observation, lesion detection, and therapeutic functions will be integrated in future stages.

Track change:

Discussion

Added future integration of observation and therapy

While the present study focuses on autonomous colonoscope insertion, complete colonoscopy involves additional steps, including mucosal inspection, lesion detection, and therapeutic intervention. Following the establishment of reliable insertion across broader anatomical conditions, the system will be extended to controlled withdrawal with systematic mucosal inspection by integrating the EOR platform's mechanical capabilities with vision-based AI. Therapeutic functions may subsequently be incorporated. In a potential clinical workflow, the operator would supervise autonomous insertion and assume a more active role during withdrawal to validate findings and perform interventions. These functions will be developed in a stepwise manner after robust insertion has been achieved. <u>These descriptions were included in page 17 line 13-22.</u>

Comment 2:

Loop formation and simulator limitations…

Response:

We acknowledge this limitation. Pattern 1 was intentionally selected for initial feasibility evaluation without looping complexity. We have clarified this and added plans for future validation in more complex models.

Notably, the EOR system actually records multiple parameters synchronized with video. We added analyses of operational parameters (force, angulation, torque), AI decision corrections (backward movements). We will explore patterns 2 through 6 similarly in the future.

Track change:

Discussion

Added explanation of Pattern 1 as a first-step feasibility model

Colonoscope insertion remains the most technically challenging phase of colonoscopy. By focusing specifically on insertion control, the present work addresses a fundamental bottleneck in the development of autonomous colonoscopy systems. Observation and lesion detection tasks, which are comparatively structured visual recognition problems, may be integrated into the system in subsequent stages after stable insertion control has been established. Our primary objective in the current study was to determine whether the AI could reliably learn the fundamental behaviors required for successful cecal intubation under controlled conditions. Therefore, we used Pattern 1 of the Kyoto Kagaku colonoscopy model as a first feasibility step. Pattern 1 was chosen deliberately to focus on whether the system can learn the critical maneuvers for reaching the cecum without the added complexity of looping or torsion. <u>These descriptions were included in page 14</u>

line 24- page 15 line 8.

Added future validation in complex colon configurations

An important feature of the present study is the stepwise translational development strategy underlying the ACRS platform. The current work represents the initial phase of this pathway, focusing on the feasibility of autonomous insertion in a controlled colon model. Future studies will extend the system to more complex colon configurations and incorporate training data from multiple endoscopists to enable the AI to learn strategies for loop formation and reduction. These descriptions were included in page 17 line 4- 8.

Comment 3:

Clarification of Levels of Autonomy…

Response

We have clarified both the standard definitions of Level 3 and Level 4 and our task-specific operational definitions used in this study.

Track change:

Methods

Revised LoA description and added clarification of definitions

Figure

Added corresponding figure: Figure 5.

Reviewer 4

We thank the reviewer for the critical perspective on clinical relevance, realism, and safety.

Comment 1:

Lack of clinical perspective and translational relevance

Response:

We agree that clinical impact is the ultimate goal. This manuscript presents an engineering proof-of-concept, but we believe it is a valuable step toward clinical translation. As noted in the Introduction, there is a global shortage of skilled endoscopists and autonomous systems could help address this gap. In this study we demonstrate that ACRS can achieve automated insertion with high success (86.1% Level-4 success). These results suggest the *potential* for clinical application of fully automated colonoscopy. We will emphasize in the revised Discussion that this is an early-stage feasibility study. We will outline the concrete translational roadmap: after simulator tests, we will proceed to preclinical animal studies and then to first-in-human trials under close supervision. By

framing this work as "preclinical validation" and specifying the necessary next steps (pig models, ethics/legal review, etc.), we will align better with the journal's focus on translational medicine.

Track change:

Discussion

An important feature of the present study is the stepwise translational development strategy underlying the ACRS platform. The current work represents the initial phase of this pathway, focusing on the feasibility of autonomous insertion in a controlled colon model. Future studies will extend the system to more complex colon configurations and incorporate training data from multiple endoscopists to enable the AI to learn strategies for loop formation and reduction. Subsequent validation in ex vivo porcine colon models followed by in vivo animal experiments will further evaluate the safety and robustness of the system before exploratory clinical studies. Such a staged development framework may facilitate the safe translation of autonomous endoscopic robotics into clinical practice. <u>These descriptions were included in page 17 line 4- 12.</u>

Comment 2:

Oversimplified colon model and lack of realistic insertion difficulty

Response:

We fully acknowledge that Pattern 1 is an oversimplified anatomy. It was chosen for this initial investigation to isolate the core challenge of learning how to advance without patient pain or loss of view. We plan to extend validation to the more difficult Kyoto Kagaku models (Patterns 2–6) which introduce redundant loops and a longer sigmoid. We agree that demonstrating insertion through tortuous anatomy is necessary for clinical realism, and will incorporate experiments on those models prior to any human trials.

Track change:

Discussion

Added explanation of Pattern 1 as a first-step feasibility model

Colonoscope insertion remains the most technically challenging phase of colonoscopy. By focusing specifically on insertion control, the present work addresses a fundamental bottleneck in the development of autonomous colonoscopy systems. Observation and lesion detection tasks, which are comparatively structured visual recognition problems, may be integrated into the system in subsequent stages after stable insertion control has been established. Our primary objective in the current study was to determine whether the AI could reliably learn the fundamental behaviors required for successful cecal intubation under controlled conditions. Therefore, we used Pattern 1 of the Kyoto Kagaku

colonoscopy model as a first feasibility step. Pattern 1 was chosen deliberately to focus on whether the system can learn the critical maneuvers for reaching the cecum without the added complexity of looping or torsion. These descriptions were included in page 14 line 24- page 15 line 8.

Added future validation in complex colon configurations

An important feature of the present study is the stepwise translational development strategy underlying the ACRS platform. The current work represents the initial phase of this pathway, focusing on the feasibility of autonomous insertion in a controlled colon model. Future studies will extend the system to more complex colon configurations and incorporate training data from multiple endoscopists to enable the AI to learn strategies for loop formation and reduction. These descriptions were included in page 17 line 4- 8.

Comment 3:

Absence of shaft rotation and unrealistic manipulation patterns

Response

It is true that in our Pattern 1 trials the expert operator did not perform extensive axial rotation of the scope – primarily because the model allowed advancing to the cecum with minimal rotation or complex maneuvers. In practice, experienced endoscopists do use rotation routinely in patients, but Pattern 1 did not require it. Our system is fully capable of shaft rotation and we will include that in future teaching data. In fact, the training data we collected included torque applied during scope rotation as one of the sensor inputs. We will clarify in the revision that because the expert could reach the cecum in the simple model without rotation, the current AI learned mostly forward advancement and tip angulation. We plan to retrain the AI with data from models requiring rotation, so that ACRS can learn realistic navigation. This point will be acknowledged as a limitation of the current model and the future need to incorporate axial rotation.

Track change:

Results (Operational parameter analysis)

Added rotational torque findings

Discussion

Added interpretation of minimal rotation

Interestingly, rotational manipulation was minimal in the AI system compared with both experts and trainees, indicating that the AI relied primarily on controlled angulation and forward advancement rather than excessive shaft rotation. These descriptions were included in page 16 line 15-19.

Comment 4:

Insufficient safety evaluation

Response

We agree that the resin model cannot replicate living tissue properties (stress–strain, perfusion, risk of perforation). In our current study, meaningful assessment of mucosal injury is not possible. We clarified the implemented safety mechanisms (force limits, torque limits, speed control, manual override, emergency stop) and added a future validation plan including ex vivo and in vivo studies.

Track change:

Discussion

Expanded description of safety features

The ACRS incorporates multiple safety layers designed to ensure safe operation, including force-controlled actuation, an insertion force limit of ±20 N, an angulation torque limit of 0.5 Nm, and speed control across all operational axes. Furthermore, the system allows real-time manual override, is equipped with an emergency stop mechanism, and enables rapid conversion to conventional manual colonoscopy when necessary. <u>These descriptions were included in page 17 line 23- page 18 line 2.</u>

Added future safety validation steps

Future studies will extend the system to more complex colon configurations and incorporate training data from multiple endoscopists to enable the AI to learn strategies for loop formation and reduction. Subsequent validation in ex vivo porcine colon models followed by in vivo animal experiments will further evaluate the safety and robustness of the system before exploratory clinical studies. Such a staged development framework may facilitate the safe translation of autonomous endoscopic robotics into clinical practice. <u>These descriptions were included in page 17 line 6- 12.</u>

All reviewer comments have been carefully addressed. The revised manuscript now provides improved methodological transparency, expanded evaluation, and a clearly defined translational pathway.

We sincerely appreciate the reviewers' valuable feedback and hope that the revised manuscript is now suitable for publication in *Communications Medicine*.

Point-by-Point Response to Reviewers (with tracked changes)

Dear Editor and Reviewers,
We sincerely thank the Editor and reviewers for their thoughtful evaluation and constructive comments. All revisions are indicated in the marked-up manuscript. Below, we provide a detailed, point-by-point response, with explicit references to where changes have been made.

Reviewer 1

Comment:

The issues have been addressed in the rebuttal, I am happy with these changes.

Response:

We thank Reviewer 1 for the positive reassessment of our revised manuscript. We are grateful that the previous concerns were considered to have been addressed.

Reviewer 2

Comment:

Authors were able to address all shortcomings of the previous version. I have no further comments to be dealt with.

Response:

We thank Reviewer 2 for the positive comments and for confirming that the shortcomings of the previous version have been addressed.

Reviewer 4

Comment:

As I noted in my previous review, the current study is still based on results obtained under a highly simplified experimental setting. At a minimum, further validation in animal

models would strengthen the translational value of the work. Even if a training model is used, evaluation under conditions more closely resembling the human colon (especially the sigmoid colon) setting would be desirable. The present setup appears to involve only limited colonoscope-specific maneuvering and may therefore be insufficient, even as an initial feasibility assessment.
In the revised version, no substantial additional validation has been provided, and I am not fully convinced that this concern has been adequately addressed. Overall, the work still seems to remain closer to an engineering proof-of-concept than to a translational research.

Response:
We thank the reviewer for this important and constructive comment. We agree that the present study was conducted under a highly simplified experimental setting and that further validation in models more closely resembling the human colon, particularly the sigmoid colon, is essential before the system can be considered translationally mature.

In the revised manuscript, we have therefore reframed the study more explicitly as an engineering proof-of-concept. We now state that the current validation was performed using Pattern 1 of a standardized colonoscopy training model and that this setting does not reproduce several key challenges of clinical colonoscopy, including redundant sigmoid anatomy, loop formation and reduction, torsion, peristalsis, patient movement, luminal contamination, variable insufflation, and in vivo tissue–scope interaction.

We also agree that animal-model validation would strengthen the translational value of the work. However, such validation was beyond the scope of the present revision. Rather than overstating the current findings, we have now clearly described this as a major limitation and have added a stepwise validation pathway as the next stage of development. This pathway includes testing in more complex colonoscopy model configurations, especially those requiring sigmoid loop management, followed by ex vivo porcine colon experiments, in vivo animal experiments, and ultimately carefully supervised early clinical evaluation.

We have also clarified that the present setup required only limited colonoscope-specific maneuvering compared with clinical colonoscopy. Therefore, the current results should not be interpreted as evidence that the system can already perform autonomous insertion in complex human colonic anatomy. Instead, they demonstrate that autonomous insertion behavior can be learned and executed by the ACRS under controlled conditions. We

believe that this more conservative framing addresses the reviewer's concern and more accurately defines the contribution and limitations of the present work.

Track change:

Discussion

It is especially important to emphasize that our study should be interpreted as an engineering proof of concept, not as a complete clinical application validation study. The validation was performed using Pattern 1 of a standardized colonoscopy training model, which does not reproduce the full anatomical and physiological complexity of clinical colonoscopy. In particular, this model does not adequately simulate redundant sigmoid anatomy, loop formation and reduction, torsion, peristalsis, patient movement, variable insufflation, luminal contamination, or in vivo tissue–scope interaction. Moreover, insertion in this configuration requires only limited colonoscope-specific maneuvering compared with difficult clinical cases. Therefore, the present findings demonstrate that the ACRS can acquire and execute basic autonomous insertion behavior under simplified conditions, but they do not establish readiness for clinical translation. Further validation will require a stepwise developmental pathway, including testing in more complex colonoscopy model configurations, especially those involving sigmoid loop formation, followed by ex vivo porcine colon experiments, in vivo animal studies, and carefully supervised exploratory clinical studies in selected low-risk cases. <u>These descriptions were included in page 19 line 4- 17.</u>

Conclusion

In conclusion, this study demonstrates the proof-of-concept feasibility of autonomous colonoscope insertion using an AI-based robotic platform trained on expert operational data. Under simplified and highly controlled model conditions, ACRS achieved a high rate of fully automated insertion and showed insertion times comparable to those of trainees. However, the present findings should not be interpreted as evidence of clinical readiness, because the current validation did not reproduce the anatomical and physiological complexity of human colonoscopy, including redundant sigmoid anatomy, loop formation, luminal contamination, and in vivo tissue–scope interaction. Further stepwise validation in more complex colon models, ex vivo and in vivo animal experiments, and carefully supervised exploratory clinical studies will be required before translational or clinical application can be considered. <u>These descriptions were included in page 19 line 13- page20 line 2.</u>

Abstract

Background: In the field of colonoscopy, robotic systems have been developed to support or replace human operators due to a shortage of trained endoscopists. We developed the Autonomous Colonoscope Robot System (ACRS), based on the Endoscopic Operation Robot version 4, to evaluate whether expert-derived operational data can enable autonomous colonoscope insertion.

Methods: ACRS was trained using insertion data obtained from an expert endoscopist operating a standardized colonoscopy training model. Automated insertions were evaluated using Pattern 1 of the model, a highly controlled configuration without substantial loop formation. Completely automated insertions were designated Level 4, whereas insertions requiring some manual assistances were designated Level 3. Level 4 insertion times were compared with manual insertions performed by an expert and trainees.

Results: Of the 72 automated insertions at Level 3 or higher, 62 are classified as Level 4, giving a success rate of 86.1% (95% CI, 75.9–93.1%). The average insertion time for Level 4 procedures is 2.92 ± 1.20 minutes, significantly longer than that of the expert (1.42 ± 0.31 minutes), but comparable to the time taken by trainees (2.94 ± 1.34 minutes).

Conclusions: ACRS demonstrates proof-of-concept feasibility for autonomous colonoscope insertion under simplified, controlled model conditions. Further validation in more complex models, animal studies, and clinical settings is required before translational application. These descriptions were included in page 2 line 1- 19.

All reviewer comments have been carefully addressed.

We sincerely appreciate the reviewers' valuable feedback and hope that the revised manuscript is now suitable for publication in *Communications Medicine*.
